# Nation-wide epidemiological study in Japan on HTLV-1 associated myelopathy/tropical spastic paraparesis using HAM-net, a novel patient registration system

**DOI:** 10.1186/1742-4690-11-S1-P17

**Published:** 2014-01-07

**Authors:** Ariella LG Coler-Reilly, Michiyo Hashimoto, Naoko Yagishita, Tomoo Sato, Hitoshi Ando, Junji Yamauchi, Natsumi Araya, Miyako Kimura, Yoshihisa Yamano, Ayako Takata

**Affiliations:** 1 Department of Rare Diseases Research, Institute of Medical Science, St. Marianna University School of Medicine, Kawasaki, Kanagawa, Japan; 2 Department of Preventive Medicine, St Marianna University School of Medicine, Kawasaki, Kanagawa, Japan

## 

HTLV-1 associated myelopathy/tropical spastic paraparesis (HAM/TSP) is a debilitating neurodegenerative disease with no known cure. Due to its rarity, there is a shortage of the information necessary to develop new treatments. Accordingly, we have built a HAM/TSP patient registration system known as HAM-net, which enabled us to gather clinical information from 304 patients throughout Japan and perform nation-wide epidemiological analysis. The patients averaged at 62.4 years old at the time of registration, 44.2 at disease onset, and 51.4 at diagnosis, with an average of 7 years between onset and diagnosis. The male-female ratio was 1:2.8. As many as 9.5% of the patients (29 individuals from 27 families) had a family history of HAM/TSP, and 6.6% of adult T-cell leukemia (ATL). Those with a family history of HAM/TSP tended to report significantly earlier ages of onset. There was enormous variation among patients in terms of the rate of motor disability development. Overall, patients required a cane at a median of 8 years post-onset, required a walker at 13 years, and completely lost the ability to walk at 18 years. However, in some patients, the symptoms progressed much more rapidly, and these patients tended to experience the worst symptoms in the end. At only 2 years post-onset, a group of 18.8% of patients already required the use of a cane, and a group of 5.5% required a walker. With these results, we have demonstrated the utility of this novel strategy, and we look forward to globalizing our study through international cooperation.

